# Identifying Risk Factors for Lower Extremity Artery Disease (LEAD) in Cardiology Patients: The Role of Ankle-Brachial Index Measurement

**DOI:** 10.3390/jcm13247858

**Published:** 2024-12-23

**Authors:** Bartosz Zambrzycki, Michał Łuczaj, Marlena Dubatówka, Karolina Dańkowska, Katarzyna Nowicka, Małgorzata Knapp, Anna Szpakowicz, Karol Kamiński, Anna Lisowska

**Affiliations:** 1 Department of Cardiology and Internal Medicine with Cardiac Intensive Care Unit, Medical University of Białystok, ul. M. Skłodowskiej-Curie 24A, 15-276 Białystok, Poland; ba.zambrzycki@gmail.com (B.Z.); michalluczaj098@wp.pl (M.Ł.); dankowska.karola@gmail.com (K.D.); knowicka171@gmail.com (K.N.); malgo33@interia.pl (M.K.); akodzi@poczta.onet.pl (A.S.); fizklin@wp.pl (K.K.); 2 Department of Population Medicine and Lifestyle Diseases Prevention, Medical University of Białystok, 15-269 Białystok, Poland; marlena.dubatowka@umb.edu.pl

**Keywords:** atherosclerosis, lower extremity arterial disease, peripheral artery disease, risk factors, ankle-brachial index, epidemiology

## Abstract

**Background and aims:** Lower Extremity Artery Disease (LEAD) is a predictor of atherosclerotic plaques in other locations and significantly increases the risk of death from cardiovascular events. This study aimed to identify cardiology patient subpopulations that should undergo Ankle-Brachial Index (ABI) measurement. **Methods:** A total of 800 patients hospitalized in the Department of Cardiology were included. Inclusion criteria were age over 40 years for men and over 45 years for women, with the ability to measure ABI. **Results:** The study group was divided into two subgroups based on ABI values, with LEAD (ABI ≤ 0.9) detected in 61 patients (7.6%). Among these, 45% exhibited symptoms of intermittent claudication. LEAD was significantly more common in patients with a lower ejection fraction, a history of myocardial infarction, coronary artery disease, coronary atherosclerosis, heart failure, hypercholesterolemia, diabetes, and in those with a past diagnosis of atherosclerosis. There was no statistical association with the incidence of ischemic stroke, renal failure, hypertension or a family history of cardiovascular disease. Average living conditions and financial status increased LEAD likelihood (*p* = 0.029; *p* = 0.018), while physical activity reduced it (*p* < 0.001). LEAD occurred more often in both current and former smokers. Patients with LEAD were more likely to be on statin therapy (*p* = 0.002). Higher hemoglobin A1c levels significantly increased the risk of LEAD. **Conclusions:** Identifying patients with risk factors for LEAD suggests that ABI measurement should be performed to detect LEAD early and implement appropriate diagnostic and therapeutic strategies.

## 1. Introduction

Peripheral artery disease (PAD) is a chronic progressive disease associated with the formation of atherosclerotic plaque in the peripheral arteries which leads to partial or complete occlusion of blood flow through the vessel. It is assumed that the disease affects approximately 230 million people worldwide, and the number of patients continues to rise. Recent evidence suggests that the prevalence of PAD has increased from ≈163 million cases in 2000 to ≈236 million in 2015. This means that from 2000 to 2015, the incidence of PAD increased globally by ≈45% [1]. The reason for this is increased life expectancy and exposure to risk factors, among which the most commonly stated are smoking, diabetes, hypertension, male sex, abnormal BMI, advanced age and certain types of dyslipidemia [2]. Patients with PAD in form of carotid atherosclerosis (CA) or the lower extremities atherosclerotic disease (LEAD) are at high risk for major adverse cardiovascular events. Additionally, the lower extremities atherosclerotic disease is a common predictor of obstructive atherosclerotic plaques occurrence in other locations such as the cerebral or coronary arteries. In the REACH (Reduction of Atherothrombosis for Continued Health) Registry, up to 61.5% of patients with LEAD had concomitant atherosclerotic disease in other arteries [3]. Patients with LEAD also have an increased risk of death from ischemic heart disease and stroke. The risk compared to patients without LEAD is five times higher in patients with asymptomatic disease and eleven times higher in symptomatic patients [4]. A common symptom of LEAD is intermittent claudication (IC), characterized by leg pain or fatigue during walking, although it is important to highlight that over 50% of patients are asymptomatic [1]. Moreover, symptoms may be masked by comorbidities, especially in elderly patients with multimorbidity, which is conducive to overlooking a diagnosis of LEAD during an examination [5].

There are several methods of diagnosing LEAD, which include walking tests, such as the treadmill test and six-minute walk test, ultrasound imaging of the arteries using the duplex technique, the ankle-brachial pressure index (ABI), and sometimes the toe-brachial index (TBI) measurement [6]. The ABI is the ratio of systolic blood pressure measured at the ankle to the highest systolic blood pressure in the arm. A result of ≤0.90 is widely used in both clinical practice and epidemiological studies to diagnose LEAD in both symptomatic and asymptomatic patients, and indicates the presence of atherosclerosis in the leg, and despite having a lower diagnostic sensitivity than duplex ultrasonography (resting ABI test sensitivity ranging from 68–84% and specificity from 84% to 99%), ABI has been recommended by the 2016 American Heart Association/American College of Cardiology (AHA/ACC) guidelines as the initial diagnostic tool for the management of patients with LEAD [7]. The advantages of the test are most certainly its usefulness in the clinical setting, as it can be easily performed in daily clinical practice at the patient’s bedside; moreover, it is an inexpensive test and does not require complex training to perform. Despite the many benefits of ABI, the disadvantages must also be mentioned. It is a test that requires at least 10 min of rest for the patient before it is performed. The test may also not detect mild or moderate disease due to the development of collateral circulation. In addition, if the patient is of advanced age, has diabetes, chronic kidney disease or has connective tissue disease, the test result may be artificially overestimated because these conditions cause calcification and stiffening of the arteries, which directly affect the result of the blood pressure measurement [8]. Furthermore, according to some studies, this method is performed infrequently or incorrectly in general practice mainly due to the lack of education and training of a standardized methodology to properly perform the measurement [9,10,11].

Numerous studies have shown that both low (≤0.90) and high (>1.40) ABI values are associated with all-cause and cardiovascular mortality. In those with an ABI between 0.81 and 0.90, total mortality was doubled, and in those with an ABI ≤ 0.70 it was four times higher [12]. It has been proven that within 5 years, 10–15% of patients with LEAD, both asymptomatic and symptomatic, in the form of intermittent claudication or any other leg pain will die, and it must be emphasized that 75% of individuals in this group will die from cardiovascular events and not due to complications related to lower extremity ischemia [13]. A study investigating the prevalence and clinical implications of abnormal, asymptomatic ankle-brachial index (ABI) in patients with significant coronary artery disease (CAD) found that many CAD patients exhibit undiagnosed LEAD as indicated by abnormal ABI values [14]. The cause of 80–90% of lower limb amputations in developed countries is impaired blood supply due to vascular disease and diabetic foot syndrome [15]. According to data from Poland’s Central Statistical Office, the number of lower limb amputations has remained unchanged since 2016. Considering the numerous consequences of LEAD, such as reduced quality of life, limb ischemia leading to amputation or disability, and increased mortality, it is essential to accurately detect and treat the disease early in at-risk patients to prevent irreversible outcomes. Therefore, our goal was to investigate in which subpopulation of cardiological patients ABI should be measured.

## 2. Materials and Methods

### 2.1. Study Population

The study group consisted of consecutive patients hospitalized in the Department of Cardiology and Internal Medicine from March 2021 to February 2024. A total of 800 people were studied (mean age 69.6 ± 10.6 years). The study group consisted of 457 men (57.1%) and 343 women (42.9%). The mean age of the male participants was 68.1 ± 10.9 (age range 40–93 years), while the mean age of female participants was 71.5 ± 9.9 (age range 47–92 years).

Inclusion criteria were an age of over 40 years in men and 45 years in women, and the ability to measure blood pressure in both upper and lower extremities to calculate the ABI of each patient. According to the 2017 ESC guidelines, ABI measurement is recommended as a non-invasive first-line test for screening and diagnosis of PAD. The test should be conducted in asymptomatic patients at risk of LEAD aged < 65 years with cardiovascular risk factors, and in patients aged > 65 years without high cardiovascular risk [16]. However, our aim was to extend these criteria slightly by including younger patients to explore whether earlier detection in this subgroup could provide additional insights into the progression and risk factors associated with peripheral artery disease. The selection of the group was random—each person meeting the inclusion criteria who came for planned hospitalization due to cardiovascular causes and had given consent to participate in the study received an identical questionnaire which he or she filled out independently or with assistance in the case of a patient’s vision difficulties, impaired movement or misunderstanding of questions, and was subjected to identical examinations. Factors excluding the patient from participating in the study, in addition to lack of consent, were limitations in performing ABI measurements: healing ulcers or wounds on the lower limbs, and inflammatory or trophic skin lesions preventing the insertion of the blood pressure cuff. The duration of the study was prolonged due to the COVID-19 pandemic, during which only life-threatening conditions were treated in our department, and patients with chronic cardiac diseases were not hospitalized at that time.

Power analysis was used to calculate appropriate sample size (total sample size = 128 participants; alpha = 0.05; power = 0.8). The number of patients enrolled (800 patients) and analyzed in terms of PAD diagnosis (61 patients with ABI > 0.9) exceeded the suggested sample size.

### 2.2. Data Collection

A proprietary questionnaire on both awareness and prevalence of cardiovascular risk factors was conducted and filled out by each patient subjectively and individually. The questionnaire consisted of 63 questions, including the patient’s medical history and ongoing health problems, regularity of medication intake, family medical history, the patient’s current symptoms that may be associated with LEAD, and questions about social conditions, physical activity and diet. The questionnaire was verified for clarity and relevance of the questions by specialists in the field of cardiology.

The validity of the selected answers was checked based on the patient’s medical history included in the hospital system only in the section regarding the patient’s current and past diseases. In addition, during the process of filling out the questionnaire, the physician was present and clearly explained the matter of the questions in the survey, and answered any questions or doubts that the patient might raise.

Medical data of the patient’s current hospitalization, including the primary reason for hospital admission, medications taken, results of measurements, laboratory tests results, outcomes of examinations performed at the Cardiology Department and diagnoses made at the end of hospitalization, were collected from the hospital system and also used for the study.

The ABI value was measured for each lower extremity in all patients using the oscillometric method (MESI ABPI MD, MESI, Ltd., Ljubljana, Slovenia). Patients were instructed to have at least 10 min of rest in a supine position before the test was conducted. The result for each individual was calculated by taking both ankle systolic pressure measurement values and then the results were divided by the highest arm systolic pressure measurement value chosen from both measurements in the upper extremities. Pathological ABI was defined as ≤0.90, measured on at least one lower extremity.

Blood pressure (BP) was measured by the oscillometric method on the same device as the ABI measurements.

Echocardiography was performed using ultrasound Vivid 9 (GE Healthcare, Chicago, IL, USA) to assess left ventricular ejection fraction (EF).

Peripheral blood was collected for laboratory testing in each fasting patient in the clinical setting. The concentrations of N-terminal pro-brain natriuretic peptide (NT-proBNP) were marked by the electrochemiluminescence method on the Cobas E411 (ROCHE Diagnostics International Ltd., Rotkreuz, Switzerland). The lipid profile was determined on the Cobas C111 (ROCHE) using the enzymatic-colorimetric method. Glucose concentration was marked by the enzymatic method with hexokinase on the same device. The fraction of glycated hemoglobin (HbA1c) was determined on D-10 (Bio-Rad Polska Sp. z.o.o., Warsaw, Poland). Serum creatinine concentration was determined on Alinity C (Abbot, Green Oaks, IL, USA) and estimated glomerular filtration rate (eGFR) was also calculated using the MDRD formula.

### 2.3. Statistical Analysis

The mean values and standard deviations for quantitative variables and the quantitative and percentage distribution for qualitative variables were calculated. Data were presented as means (%) and standard deviation (SD) distributed continuous variables, medians (Me) and interquartile range (IQR) for not normally distributed continuous variables, and as the number (N) of cases and percentages (%) for categorical variables. Comparisons of variables between subgroups were conducted using the Mann–Whitney, Kruskall–Wallis, or Fisher’s tests. Associations between the ABI and other clinical and biochemical variables were analyzed using a multiple and stepwise backward logistic regression model adjusted for age, gender and BMI. Statistical hypotheses were verified at a 0.05 significance level. IBM SPSS Statistics 26.0 statistical software (Armonk, NY, USA) was used for all calculations.

The study was conducted in accordance with the Declaration of Helsinki and approved by the Ethics Committee of the Medical University of Bialystok (Poland) (approval number: R-I-002/565/2018, 29 November 2018).

## 3. Results

### 3.1. Study Group Characteristics

**Figure 1:**
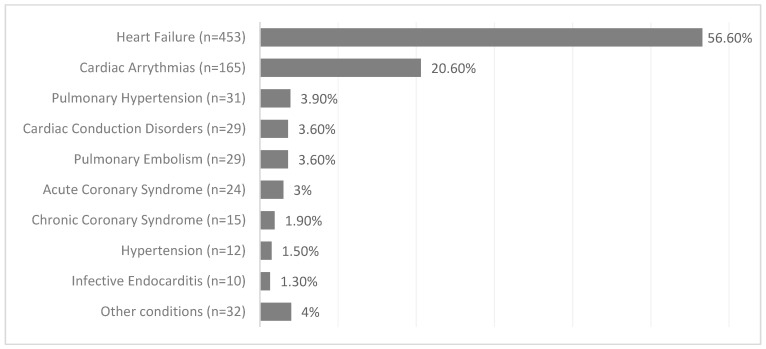
The primary reason for hospitalization in the study population.

The primary reason for hospitalization of patients admitted to the Cardiology Department is illustrated in the Figure 1. As a result of the conducted diagnostics, heart failure was found in 588 (74.3%). 583 (72.9%) patients had hypertension, 438 (54.6%) had atrial fibrillation (AF), and 300 (37.5%) had coronary artery disease (CAD).

**Figure 2:**
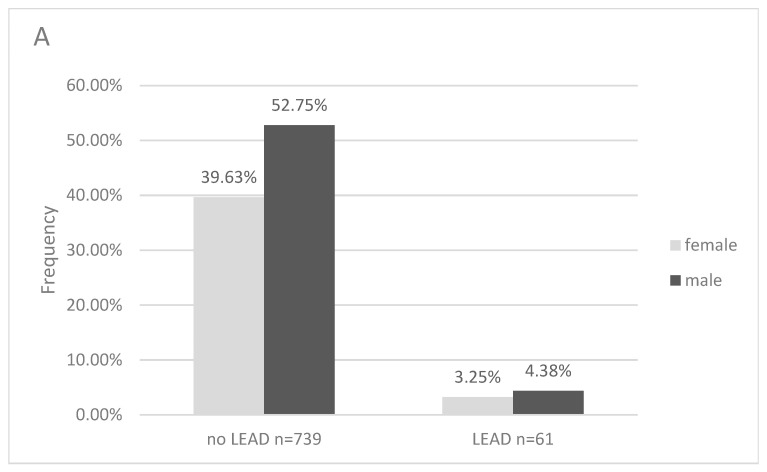

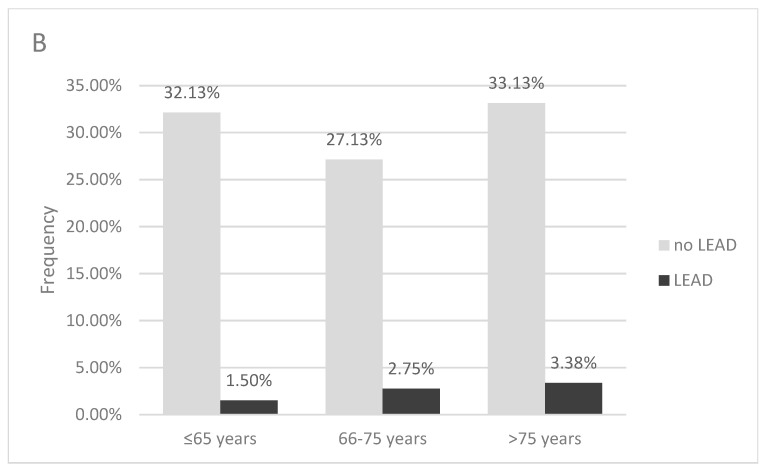
Presence of LEAD in the study population (n = 800) taking into account gender (**A**) and age (**B**).

The study group (n = 800) was divided into two subgroups depending on the presence of lower extremities atherosclerotic disease diagnosed based on the ABI value. An ABI value ≤ 0.9 detected on at least one lower extremity was considered abnormal according to the ESC and ESVS guidelines [16]. LEAD was found in 61 patients, which was 7.6% of the study group (Figure 2). In the youngest age group of individuals between 40–65 years, the presence of PAD was 12 (1.5%) patients—female 0.2%, male—1.3%. Among those aged 66–75 years the presence of LEAD was 22 (2.8%) patients (F—1.5%, M—1.3%), and in the oldest age group of individuals > 75 years old was 27 (3.4%) patients (F—1.5%, M—1.9%).

The average ABI value in men was 1.12 (±0.13) for the left, and 1.13 (±0.15) for the right lower extremity, and in women it was 1.11 (±0.11) for the left, and 1.12 (±0.12) for the right lower extremity.

Of the 61 patients with reduced ABI value (≤0.9), 50 (82%) reported pain when walking, of which 27 (44.3%) had typical symptoms of intermittent claudication.

Patients considered to have typical symptoms of intermittent claudication are those who indicated in the questionnaire that they experience discomfort and pain in their legs when walking; the pain mainly occurs during brisk walking and when climbing uphill or stairs, subsides within 10 min with rest, and occurs in the typical location. The pain occurs daily or several times a week depending on the frequency of physical activity undertaken by the patient and is reproducibly triggered by a specific same physical exertion. Of the patients experiencing similar symptoms, 13 also had symptoms at rest that worsened during movement.

Seven patients (11.4%) reported a history of ulcers on the toe, foot or shin that healed slowly or not at all, and 19 patients (31.1%) regularly perceived that one lower limb or foot was subjectively colder compared to the other lower limb.

A total of 397 (49.6%) patients, of whom 292 (36.5%) were men and 105 (13.1%) were women, declared to be smokers (current or past). The group of active smokers included 104 (13%) patients.

Regarding medication use, 610 (76.3%) patients were taking statins, 187 (23%) patients were on antiplatelet therapy (acetylsalicylic acid), 468 (58.5%) patients were taking angiotensin-converting-enzyme inhibitors (ACEI), 186 (23.3%) patients were taking angiotensin receptor blockers (ARB), 221 (27.6%) patients were taking calcium channel blockers (CCB), and 595 (74.4%) patients were taking diuretics.

### 3.2. Lower Extremities Atherosclerotic Disease (LEAD)

The study group (n = 800) was divided into 2 subgroups depending on the presence or absence of LEAD on the basis of ABI values measured for each patient, and the characteristics of these subgroups are presented in Table 1.

#### 3.2.1. Interview and Examination Data

Patients who were diagnosed with LEAD were significantly older compared to the control group (*p* = 0.003). There was no statistically significant association with gender. Patients with LEAD had a higher BMI value (*p* = 0.047) and a statistically significant lower left ventricular ejection fraction (*p* = 0.004). Almost 60% of patients with ABI ≤ 0.9 reported lower limb pain after walking less than 100 m, and only one patient in this group reported a walking distance of more than 500 m.

#### 3.2.2. Past Medical History

Patients with LEAD were affected significantly more frequent by diseases of atherosclerotic etiology such as myocardial infarction (*p* < 0.001), CAD (*p* < 0.001) and carotid atherosclerosis (*p* < 0.001). In addition, these patients were more often diagnosed with heart failure (*p* < 0.001), renal failure (*p* < 0.025), ischemic stroke (*p* < 0.041), and diseases that directly contribute to the development of atherosclerosis, which are diabetes (*p* = 0.001) and hypercholesterolemia (*p* = 0.002). There were no differences in the presence of LEAD in patients with hypertension and those with normal blood pressure.

#### 3.2.3. Socio-Economic Status

It was observed that patients with average living conditions and financial situations contributed to a higher incidence of LEAD (*p* = 0.029; *p* = 0.018); whereas for people with better living conditions and financial situations, LEAD occurred at a lower rate.

#### 3.2.4. Lifestyle Factors

Engaging in physical activity had a substantial effect on reducing the incidence of LEAD among examined patients (*p* < 0.001). The frequency of physical activity has shown that performing physical activity less than once a week significantly contributes to the incidence of the disease, and daily activity does not correlate with the occurrence of the disease. Furthermore, no significant effect of the use of diet or smoking was seen in the presence of LEAD development. The data presented in the Table 1. shows that active or past smoking does not correlate with the presence of LEAD, but it was observed that the length of smoking has a significant relationship with low ABI values (R = −0.18, *p* < 0.001). The average number of pack-years for patients with LEAD was 32.3 (±24.5), and for patients without LEAD this was 23.1 (±18.8) pack-years.

#### 3.2.5. Pharmacotherapy

Patients with ABI value ≤ 0.9 were more frequently on statin pharmacotherapy than patients with normal ABI values (*p* = 0.002).

#### 3.2.6. Biochemistry

Patients with LEAD had significantly elevated levels of HbA1c and triglycerides compared to patients from the second subgroup. In addition, lower GFR and LDL-cholesterol levels were observed in patients with lower ABI. NTproBNP and fasting glucose levels did not differ for patients in each studied subgroup.

#### 3.2.7. Additional Findings

Some factors, such as the presence of a history of cardiovascular diseases occurring in the patient’s family, the difference in the patient’s educational status divided into primary and higher levels of education, and the patient’s place of residence divided into rural areas and urban areas with different population densities were found to have no significant correlation with low ABI values.

### 3.3. Multivariate Analysis of LEAD Occurrence: Model 1 Adjusted for Age; Model 2 Adjusted for Model 1 + BMI; Model 3 Adjuster for Model 2 + Current Smoking (n, %)

The next analysis (Table 2) shows the association of individual factors with the incidence of LEAD adjusted for various factors.

Multivariate analysis of LEAD occurrence adjusted for age, BMI, and current smoking showed that LEAD was significantly more common in patients with a lower ejection fraction, a history of myocardial infarction, coronary artery disease, coronary atherosclerosis, heart failure, hypercholesterolemia, diabetes, and in those with a past diagnosis of atherosclerosis. There was no statistical association with the incidence of ischemic stroke, renal failure, hypertension, or a family history of cardiovascular disease. LEAD was more common in individuals with average socioeconomic conditions. LEAD was notably less frequent in patients who reported an active lifestyle and more prevalent among those who exercised less than once per week.

Moreover, LEAD occurred more often in both current and former smokers. We did not find statistically significant differences in the incidence of LEAD according to dietary adherence. In terms of pharmacotherapy, patients diagnosed with LEAD were more likely to be on statin therapy, and there was no significant correlation between LEAD and the use of acetylsalicylic acid. Furthermore, higher hemoglobin A1c levels significantly increased the risk of LEAD.

### 3.4. Regression Analysis of Predictors of LEAD

Logistic regression analysis with stepwise variable elimination (Table 3) was performed to assess which element of past medical history best correlates with the incidence of LEAD, taking into account age, BMI, and smoking. Myocardial infarction and carotid atherosclerosis show a significant relationship with the incidence of LEAD after accounting for the previously mentioned factors.

**Figure 3:**
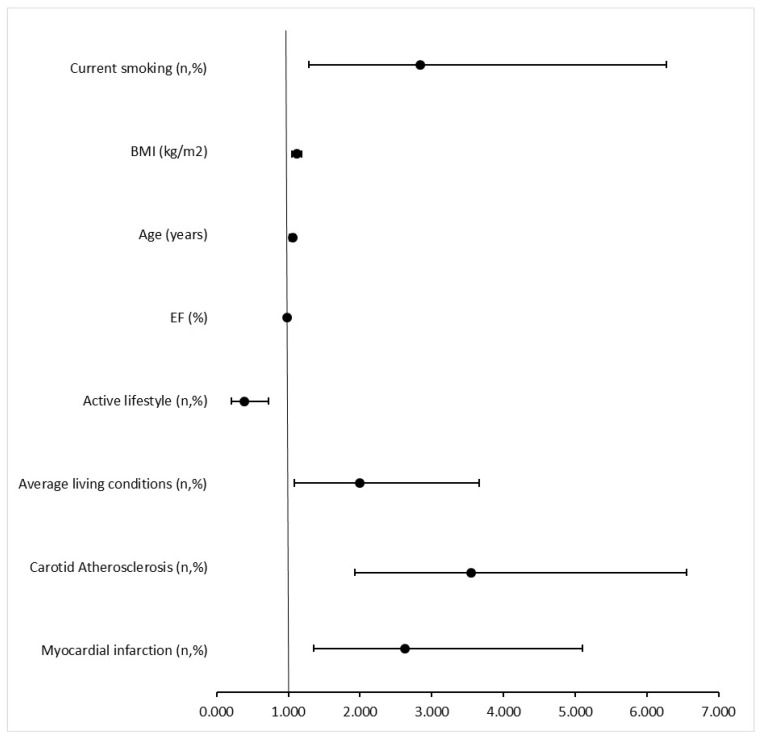
Multivariate logistic regression analysis of predictors of LEAD.

A multivariate logistic regression model (Figure 3) was performed to assess which factors significantly influence the presence of LEAD. In the study population, both a higher frequency of past myocardial infarctions (95% CI 1.350–5.107, *p* = 0.004) and the presence of atherosclerosis in the carotid arteries (95%CI 1.923–6.552, *p* < 0.000) influence the presence of LEAD. In addition to these, it is increased BMI (95%CI 1.053–1.183, *p* < 0.000), age (95%CI 1.021–1.097, *p* = 0.002), more frequent smoking (95%CI 1.288–6.276, *p* = 0.01), average living conditions (95%CI 1.081–3.666, *p* = 0.03) and lower physical activity (95%CI 0.208–0.718, *p* = 0.003) that influence the presence of LEAD.

## 4. Discussion

Patients with atherosclerosis are at an elevated risk of ischemic incidents or developing cardiovascular disease, which is the leading cause of death worldwide, despite the widespread use of primary and secondary prevention and pharmacotherapy according to the latest guidelines. It should be understood that atherosclerosis is a systemic disease, affecting more than just one vascular bed, and in addition to the peripheral vessels, atherosclerotic lesions can also involve the coronary arteries. Patients with peripheral artery disease in the form of carotid atherosclerosis (CA) or the lower extremities atherosclerotic disease (LEAD), are at high risk of major adverse cardiovascular events [17,18,19]. In addition, atherosclerotic plaque morphology changes with age. Older patients are more likely to have stable fibrotic lesions, while younger patients are more likely to have unstable lesions with a higher risk of rupture [5]. Therefore, it is necessary to direct attention to early preventive measures in the group of patients at high cardiovascular risk and to know the risk factors for ischemic incidents in the broader population of individuals with atherosclerosis, often still clinically asymptomatic.

The main objective of our study was to answer the question of in which subgroup of cardiological patients it could be beneficial to conduct an examination for the diagnosis of LEAD. By distinguishing the main risk factors that significantly increase the risk of the disease, we were able not only to detect an individual with asymptomatic disease or symptomatic and previously undiagnosed disease, but also to prevent the development of the disease in individuals with these factors by early introduction of preventive pharmacological treatment. In our study, in order to separate the subgroup of patients with LEAD, we chose the method of oscillometric measurement of ABI. Despite the NICE and ESC guidelines recommending using Doppler devices over automated ABI measurement for LEAD diagnosis [16,20], there are claims that the oscillometric method can be more beneficial in outpatient screening than the Doppler technique [21,22], especially in primary care settings [23,24,25,26,27,28]. Another definite advantage of this method is its usefulness in terms of the time needed for evaluation, which was significantly lower as opposed to other techniques with higher referentiality [29,30]. Moreover, in a study conducted by Vega et al. it was found that Doppler measurements performed by physicians not specially trained in the Doppler technique provided less accurate diagnostic accuracy in determining ABI values than performing the test using the oscillometric method by the same individuals [31,32], thus also prompting the choice of the oscillometric technique to enable diagnostics in less specialized centers for a broader range of patients.

It is worth mentioning the limitations of our study that may have affected some of the obtained results. Many studies highlight that the oscillometric technique often yields inaccurate ABI results in patients with diabetes and chronic kidney disease due to an exaggeration of the values caused by arterial stiffness arising from the calcification process, inability to detect low pressures, and general overestimation of the ABI values when compared to measurements by other techniques such as Doppler ultrasound [33,34,35,36]. Referring to the most recent guidelines, in this group of patients with non-compressible ankle arteries, a TBI (Toe-Brachial Index) measurement should be performed if the ABI measurement result is normal and also if the ABI measurement result is >1.40 [37]. In our study group, there were only 13 (1.6%) patients whose ABI value was >1.40 in at least one lower extremity; however, the primary aim of our study was to evaluate the utility of the ABI measurements as a rapid and easily accessible screening tool for LEAD, particularly when performed by minimally trained personnel. Given this focus on simplicity and feasibility in routine clinical practice, we did not pursue further diagnostic evaluation, such as Toe-Brachial Index (TBI) measurement, in patients with ABI values above 1.40. Regarding ABI measurements, it has been suggested that increasing the cutoff points to higher than 0.9 may help increase the efficiency of LEAD detection by the oscillometry method [33,38,39,40,41]. Another limitation was the inability to measure ABI in patients who had already undergone limb amputation or had non-healing leg ulcers due to critical limb ischemia, which made it impossible to perform the assessment in these cases.

In conclusion, the oscillometric method seems to be an appropriate tool for screening for LEAD on a large scale, especially in primary care settings and under the lack of specialized staff to perform more advanced diagnostic tests [36]. Unfortunately, in the realities of medical practice, this method remains undervalued or, as mentioned earlier, under-practiced [9,10,11]. A survey of Polish facilities found that 74% of medics in Polish primary care facilities consider ABI testing to be a good diagnostic method; however, many clinicians indicated that they did not have the proper equipment to perform ABI measurements and many had not acquired the skills to perform the test during their university education [42].

In our study population, we detected PAD localized in the lower extremities in 7.6% of cardiological patients, a value very consistent with data in a systematic review estimating that in high-income countries in a population of similar age, the prevalence of LEAD averages around 7.8%, and in middle-income and low-income countries around 6.4% [43]. This is also comparable to findings from the Białystok PLUS study, a population-based survey conducted in the same region as our cohort. In contrast to our study, which was focused on patients hospitalized in the Cardiology Department, Białystok PLUS assessed the general population and reported an ABI value equal or below 0.9 in 8.5% of participants, using a similar oscillometric method [44]. In our study, among 61 patients with LEAD, 50 patients answered affirmatively to a question on the questionnaire regarding the presence of lower extremity pain. Then, after analyzing further responses and excluding some patients due to pain etiology unrelated to atherosclerotic disease, it was found that 27 (45%) had the typical symptoms of intermittent claudication. This indicates that more than half of the patients remained asymptomatic, which is consistent with observations from other studies [13,17,44].

Patients diagnosed with LEAD were older and had higher BMI relative to the control group. There was no association with gender. Patients in this population were significantly more frequently diagnosed with heart failure, diabetes and hypercholesterolemia. We found no difference in the prevalence of hypertension. In the WOBASZ II study, hypertension was found to increase the risk of developing LEAD by 50%; perhaps the lack of significant difference in the prevalence of hypertension in patients with and without LEAD is due to the fact that hypertension is a very common condition in patients hospitalized in a Cardiology Department [45]. Because atherosclerosis is a progressive and systemic disease and very often does not occur in just one vascular bed, we demonstrated in our patients with LEAD a significantly higher prevalence of myocardial infarction, CAD, carotid atherosclerosis and ischemic stroke, which is in line with previous studies [3].

Lifestyle factors were also important. It is noteworthy that patients with LEAD who described their living conditions as average or poor were more likely to be ill. It is proven that good socioeconomic conditions reduce the number of potential risk factors for the development of cardiovascular disease, mainly influencing aspects such as frequency of smoking or lack of dietary adherence [46,47,48]. Physical activity significantly reduced the incidence of LEAD in the studied patients, with regular and frequent exercise being particularly impactful. Many previous studies have shown an impact on the prevention of lower limb arterial disease, especially in the asymptomatic phase. Physical exercise of at least 150 min per week reduces all-cause mortality, reduces the incidence of chronic disease, and improves quality of life [49]. Studies also have shown that frequent moderate-intensity training has a significantly greater effect on the redox system compared to irregular and intense training [50].

In our study, dietary adherence did not appear to be a significant factor in increasing the incidence of LEAD; previous studies have highlighted the positive impact of a Mediterranean diet in reducing the risk of atherosclerosis [51]. This discrepancy may be attributed to the general dietary habits of the study population which deviate from those assumed to be healthy due to the irregularity of meal intake, consumption of excessive sweets between meals, and under-consumption of vegetables, fish and whole grain products [52]. In our study, a distinct minority of patients followed any particular diet.

In contrast to many previous studies [53], being a smoker did not significantly influence the incidence of LEAD. However, an association with the duration of smoking, regardless of whether the patient was an active smoker or had quit, was observed. Additionally, an association with smoking intensity was noted, where patients with a higher pack-years index were more likely to have LEAD, aligning with previous research findings [53].

A significant body of evidence supports the notion that lifestyle modifications play a crucial role in reducing cardiovascular risk, especially in individuals classified as low-risk. This is comparable to findings from the Białystok PLUS study, which similarly identified that lifestyle modifications, when consistently applied, have substantial effects on lowering cardiovascular risk, potentially offering similar or even better outcomes compared to pharmacological interventions [54].

It should be noted that patients with a lower ABI have been shown to have more frequent biochemical abnormalities such as elevated glycated haemoglobin, triglycerides or reduced GFR. In our study group, older patients and those with a lower ABI had lower LDL-cholesterol concentrations. We suspect that this is due to the efficient and longer duration of statin pharmacotherapy. Lower GFR values in patients with LEAD are of paramount importance, as proven by previous studies demonstrating that renal failure is a significant independent predictor of asymptomatic LEAD [55]. The co-occurrence of LEAD and renal disease was the primary contributor to LEAD-related death in the 40–59 age group, especially in women. High fasting plasma glucose (FPG) is the primary risk factor for LEAD- related death too [56].

In terms of pharmacotherapy, patients with a lower ABI were significantly more likely to take statins since it is a mandatory drug in the standard therapy of patients with atherosclerosis. No significant differences were found in the uptake of acetylsalicylic acid. Statins are drugs that have significant benefits in reducing the risk of death and cardiovascular events and their importance was highlighted in the most recent 2024 ESC Guidelines in which the recommendation for lipid-lowering therapy for patients with peripheral arterial and aortic diseases was changed from I C to I A class [37]. The lack of association with ASA intake may be related to guidelines according to which antiplatelet therapy is not recommended for inclusion in patients with asymptomatic LEAD without any sign of clinically relevant atherosclerotic cardiovascular disease (class III B). ASA monotherapy is mandatory only for patients with diagnosed, symptomatic disease (class I A) [37]. Based on the randomized COMPASS Trail, the combination of rivaroxaban and ASA is recommended for patients with stable coronary artery or peripheral artery disease in whom there is no clinical indication for full-dose anticoagulant therapy [57].

To summarize the previous findings, one of the unexpected results of our study was that hypertension did not increase the risk of LEAD, but it should be mentioned that already in the previous TASC guidelines the relative risk of developing LEAD was lower in patients with hypertension than in patients with diabetes and smokers. Additionally, in our study, LDL levels were found to be low across the group, primarily due to the widespread use of statins among the participants, which were also used more frequently compared to a subpopulation of patients without LEAD. Furthermore, a family history of cardiovascular diseases and dietary factors did not show a significant association with LEAD risk. These factors highlight the importance of considering population-specific traits when interpreting the results.

It should be emphasized that a population consisting of patients hospitalized in the Department of Cardiology, as compared to the general population, has a higher cardiovascular risk profile due to the conditions that warranted their hospitalization. The high prevalence of atherosclerotic comorbidities among the study population is evidence that this is the population particularly vulnerable to LEAD. Special attention should be paid to those cardiac patients who do not have clinical symptoms of LEAD, but who also present known risk factors such as age, elevated BMI, smoking, additional risk factors for LEAD, i.e., a history of myocardial infarction, carotid atherosclerosis, poor socioeconomic conditions or low physical activity. Understanding these specific risk factors of prevalence of LEAD in a hospitalized cohort of cardiology patients should prompt consideration of whether such patients should be screened for atherosclerosis-related disease by performing a simple and not time-consuming ABI examination in order to apply early and more effective prevention. This approach can help reduce the burden of LEAD and its associated complications.

Referring to the guidelines, ABI measurement is recommended as a non-invasive first-line test for screening and diagnosis of PAD (Class I C recommendation in the ESC 2017 guidelines and Class I B in the ESC 2024 guidelines), and the test should be considered in patients aged > 65 years with cardiovascular risk factors (Class IIa C) and can be considered in patients aged > 65 years without cardiovascular risk factors (Class IIb C) [37]. However, according to our findings, we believe that performing ABI testing in a clinical setting in patients hospitalized in cardiology departments (irrespective of their age), especially in the group of patients with risk factors that affect the probability of developing LEAD, would be a good practice, considering that the diagnosis of the disease and the initiation of treatment directly affects the reduction of mortality and the comfort of the patient’s life, and the form of the test itself, due to the ease of performance and availability of the method we mentioned above, facilitates conducting it on a large scale.

There is a need for more research in this issue. To enable more precise identification of risk groups and associated factors, it would be important to conduct studies that include a much larger patient population from multiple centers. Furthermore, it would also be advisable to carry out a study involving the measurement of TBI in certain subpopulations of patients, as these studies have often had very low evidence value [58].

## 5. Conclusions

In our study, we showed that the outlier of patients with LEAD among the population of patients admitted to a Cardiology Department was 7.6%. Among the diseased patients, 55% were asymptomatic. The most potent factors influencing the occurrence of LEAD were past myocardial infarction, presence of atherosclerosis in the carotid arteries, a past or current smoking habit, average socioeconomic conditions, and low physical activity. These findings allow us to conclude that the presence of the above-mentioned factors may suggest performing a minimally invasive ankle-brachial index measurement test in order to detect LEAD early and implement effective diagnostic and therapeutic strategies to directly affect the prolongation of the patient’s life in full health, prevent future cardiovascular incidents that may lead to further hospitalizations and even death, and avoid potential complications related to lower limb ischemia.

## Figures and Tables

**Table 1:** Characteristics of the study subgroups according to the ABI value.

Variables	Patients Without LEAD—ABI > 0.9 (n = 739)	Patients with LEAD—ABI ≤ 0.9 (n = 61)	*p*-Value
**Interview and examination data**			
Age (years)	70 (62–77)	74 (68.5–81)	0.003
Gender, men (n, %)	422 (57.1%)	35 (57.38%)	0.539
BMI (kg/m^2^)	28.4 (25.35–31.83)	29.3 (25.83–33.61)	0.047
ABI left ankle	1.14 ± 0.10	0.87 ± 0.12	<0.001
ABI right ankle	1.15 ± 0.11	0.83 ± 0.12	<0.001
SBP (mmHg)	128 (117–144)	132 (115–151)	0.145
DBP (mmHg)	78 (70–85)	80 (65–86.5)	0.878
EF (%)	54.5 (40–60)	50 (31–55)	0.004
Lower limb pain after walking < 100 m	184 (24.9%)	36 (59.0%)	0.002
**Past medical history**			
Myocardial infarction (n, %)	134 (18.13%)	26 (42.62%)	<0.001
Ischemic stroke (n, %)	68 (9.20%)	11 (18.03%)	0.041
Coronary artery disease (n, %)	223 (30.18%)	37 (60.66%)	<0.001
Carotid atherosclerosis (n, %)	134 (18.13%)	32 (52.46%)	<0.001
Heart failure (n, %)	475 (64.28%)	52 (85.25%)	<0.001
Renal failure (n, %)	88 (11.91%)	14 (22.95%)	0.025
Hypercholesterolemia (n, %)	406 (54.94%)	46 (75.41%)	0.002
Diabetes (n, %)	204 (27.6%)	30 (49.18%)	0.001
Hypertension (n, %)	522 (70.64%)	47 (77%)	0.378
Aortic aneurysm (n, %)	20 (2.71%)	5 (8.2%)	0.036
Past diagnosis of atherosclerosis (n, %)	197 (26.66%)	28 (45.9%)	0.001
**Socio-economic status**			
Good financial situation (n, %)	246 (33.29%)	12 (19.67%)	NS
Average financial situation (n, %)	281 (38.02%)	32 (52.46%)	0.029
Poor financial situation (n, %)	36 (4.87%)	2 (3.28%)	NS
Good living conditions (n, %)	347 (46.96%)	20 (32.79%)	NS
Average living conditions (n, %)	204 (27.6%)	26 (42.62%)	0.018
Poor living conditions (n, %)	8 (1.08%)	0 (0%)	NS
**Lifestyle factors**			
Active lifestyle (n, %)	471 (63.73%)	23 (37.7%)	<0.001
Physical activity everyday (n, %)	258 (34.91%)	16 (26.23%)	0.204
Physical activity 3–5 times a week (n, %)	134 (18.13%)	2 (3.28%)	0.034
Physical activity 1–2 times a week (n, %)	58 (7.85%)	2 (3.28%)	0.446
Physical activity less than once a week (n, %)	15 (2.03%)	3 (4.92%)	0.047
Dietary management (n, %)	245 (33.15%)	24 (39.34%)	0.483
Current smoking (n, %)	91 (12.31%)	13 (21.31%)	0.071
Past smoking (n, %)	288 (38.97%)	29 (47.54%)	0.05
**Pharmacotherapy**			
Statins (n, %)	554 (74.97%)	56 (91.8%)	0.002
Acetylosalicic acid (n, %)	168 (22.73%)	19 (31.15%)	0.156
**Biochemistry**			
Fasting glucose (mmol/L)	5.2 (4.7–6.1)	5.3 (4.9–6.4)	0.062
Hemoglobin A1c (%)	6.1 (5.7–6.7)	6.4 (5.9–7.4)	0.023
GFR (ml/min/1.73 m^2^)	71.5 (54–89)	65 (48–81)	0.045
LDL-cholesterol (mmol/L)	2.17 (1.60–2.97)	1.72 (1.28–2.33)	0.003
NT-proBNP (pg/mL)	635.5 (219.25–1633.75)	765 (376–2059)	0.104
Triglycerides (mmol/L)	1.05 (0.80–1.42)	1.25 (0.91–1.72)	0.011

Data are presented as median (Q1–Q3) or n (%). BMI, body mass index; ABI, ankle-brachial index; SBP, systolic blood pressure; DBP, diastolic blood pressure; EF, ejection fraction; GFR, glomerular filtration rate; LDL, low-density lipoproteins; NT-proBNP, n-terminal pro b-type natriuretic peptide.

**Table 2:** Multivariable predictors of LEAD.

Variables	Model 1		Model 2		Model 3	
	OR (95%Cl)	*p*-Value	OR (95%Cl)	*p*-Value	OR (95%Cl)	*p*-Value
**Interview and examination data**						
Gender, men (n, %)	1.147 (0.671;1.962)	0.615	1.187 (0.69;2.04)	0.536	1.104 (0.629;1.936)	0.731
BMI (kg/m^2^)	1.087 (1.034;1.142)	0.001	-	-	-	-
SBP (mmHg)	1.007 (0.995;1.019)	0.25	1.007 (0.994;1.019)	0.293	1.008 (0.995;1.02)	0.229
DBP (mmHg)	1.005 (0.983;1.028)	0.647	1.003 (0.981;1.026)	0.761	1.003 (0.981;1.026)	0.783
EF (%)	0.974 (0.956;0.991)	0.003	0.972 (0.955;0.99)	0.002	0.972 (0.954;0.991)	0.003
Lower limb pain after walking < 100 m	0.44 (0.205;0.941)	0.034	0.473 (0.219;1.021)	0.057	0.439 (0.2;0.965)	0.04
**Past medical history**						
Myocardial infarction (n, %)	3.37 (1.953;5.817)	>0.001	3.687 (2.111;6.439)	>0.001	3.848 (2.18;6.794)	>0.001
Ischemic stroke (n, %)	1.902 (0.937;3.859)	0.075	1.846 (0.902;3.778)	0.093	1.793 (0.87;3.696)	0.114
Coronary artery disease (n, %)	2.885 (1.655;5.031)	>0.001	2.697 (1.542;4.719)	0.001	2.83 (1.607;4.982)	>0.001
Carotid atherosclerosis (n, %)	4.58 (2.604;8.055)	>0.001	4.494 (2.535;7.967)	>0.001	4.646 (2.602;8.297)	>0.001
Heart failure (n, %)	3.533 (1.477;8.453)	0.005	3.283 (1.367;7.883)	0.008	3.227 (1.338;7.785)	0.009
Renal failure (n, %)	1.857 (0.97;3.556)	0.062	1.611 (0.827;3.136)	0.161	1.648 (0.84;3.231)	0.146
Hypercholesterolemia (n, %)	2.539 (1.368;4.714)	0.003	2.41 (1.294;4.491)	0.006	2.717 (1.425;5.183)	0.002
Diabetes (n, %)	2.364 (1.39;4.02)	0.001	1.974 (1.138;3.424)	0.016	2.115 (1.207;3.706)	0.009
Hypertension (n, %)	1.194 (0.639;2.232)	0.578	0.977 (0.515;1.855)	0.944	0.924 (0.483;1.768)	0.811
Aortic aneurysm (n, %)	2.829 (1.012;7.906)	0.047	2.385 (0.843;6.743)	0.101	2.322 (0.809;6.667)	0.117
Past diagnosis of atherosclerosis (n, %)	2.286 (1.318;3.964)	0.003	2.353 (1.348;4.107)	0.003	2.618 (1.478;4.637)	0.001
Family history of cardiovascular diseases (n, %)	1.248 (0.687;2.265)	0.467	1.18 (0.646;2.155)	0.591	1.221 (0.661;2.258)	0.523
**Socio-economic status**						
Good financial situation (n, %)	0.503 (0.262;0.967)	0.039	0.568 (0.295;1.096)	0.092	0.612 (0.315;1.189)	0.147
Average financial situation (n, %)	1.719 (1.015;2.913)	0.044	1.704 (1.001;2.899)	0.049	1.602 (0.935;2.747)	0.087
Poor financial situation (n, %)	0.732(0.171;3.139)	0.675	0.803 (0.186;3.471)	0.769	0.757 (0.171;3.34)	0.713
Good living conditions (n, %)	0.593 (0.339;1.035)	0.066	0.963 (0.504;1.839)	0.909	0.886 (0.458;1.715)	0.720
Average living conditions (n, %)	1.809 (1.058;3.095)	0.030	1.812 (1.054;3.115)	>0.001	1.785 (1.03;3.094)	>0.001
Poor living conditions (n, %)	0.000 (0.000)	0.999	0.000 (0.000)	0.999	0.000 (0.000)	0.999
**Lifestyle factors**						
Active lifestyle (n, %)	0.353 (0.204;0.610)	>0.001	0.394 (0.225;0.687)	0.001	0.386 (0.218;0.682)	0.001
Physical activity everyday (n, %)	1.834 (0.741;4.541)	0.190	1.828 (0.73;4.578)	0.198	1.86 (0.726;4.767)	0.196
Physical activity 3–5 times a week (n, %)	0.235 (0.054;1.017)	0.053	0.238 (0.055;1.032)	0.055	0.219 (0.05;0.97)	0.05
Physical activity 1–2 times a week (n, %)	0.668 (0.153;2.925)	0.593	0.667 (0.149;2.99)	0.597	0.692 (0.151;3.179)	0.636
Physical activity less than once a week (n, %)	5.031 (1.317;19.229)	0.018	4.65 (1.205;17.953)	0.026	5.688 (1.389;23.282)	0.016
Dietary management (n, %)	1.218 (0.710;2.089)	0.474	1.154 (0.668;1.992)	0.607	1.314 (0.752;2.296)	0.337
Current smoking (n, %)	2.703 (1.356;5.387)	0.005	2.998 (1.484;6.058)	0.002	-	-
Past smoking (n, %)	2.247 (1.212;4.165)	0.01	2.203 (1.181;4.111)	0.013	2.139 (1.14;4.013)	0.018
**Pharmacotherapy**						
Statins (n, %)	3.688 (1.450;9.380)	0.006	3.345 (1.309;8.545)	0.012	3.218 (1.255;8.251)	0.015
Acetylosalicic acid (n, %)	1.476 (0.832;2.615)	0.183	1.58 (0.885;2.819)	0.122	1.636 (0.909;2.944)	0.100
**Biochemistry**						
Fasting glucose (mg/dL)	1.003 (0.997;1.010)	0.322	1.002 (0.995;1.009)	0.554	1.003 (0.996;1.01)	0.473
Hemoglobin A1c (%)	1.280 (0.989;1.656)	0.06	1.255 (0.967;1.63)	0.088	1.37 (1.04;1.804)	0.025
GFR (ml/min/1.73 m^2^)	0.995 (0.984;1.006)	0.327	0.996 (0.985;1.007)	0.463	0.996 (0.985;1.007)	0.453
LDL-cholesterol (mmol/L)	0.994 (0.986;1.001)	0.112	0.994 (0.987;1.002)	0.130	0.993 (0.986;1.001)	0.091
NT-ProBNP (pg/mL)	1.000 (1.000;1.000)	0.188	1.000 (1.000;1.000)	0.081	1.000 (1.000;1.000)	0.078

Model 1 adjusted for age; model 2 adjusted for model 1 + BMI; model 3 adjuster for model 2 + Current smoking (n, %). BMI, body mass index; ABI, ankle-brachial index; SBP, systolic blood pressure; DBP, diastolic blood pressure; EF, ejection fraction; GFR, glomerular filtration rate; LDL, low-density lipoproteins; NT-proBNP, n-terminal pro b-type natriuretic peptide.

**Table 3:** Results of stepwise backward logistic regression analysis of predictors of LEAD.

	Full Model		Final Model	
	*p*-Value	OR (95%Cl)	*p*-Value	OR (95%Cl)
Age (years)	0.009	1.053 (1.013;1.094)	0.003	1.058 (1.02;1.098)
BMI (kg/m^2^)	0.002	1.099 (1.035;1.166)	>0.001	1.111 (1.048;1.178)
Current smoking (n, %)	0.005	3.32 (1.439;7.66)	0.006	3.186 (1.405;7.221)
Myocardial infarction (n, %)	0.04	2.113 (1.033;4.321)	0.003	2.7 (1.401;5.206)
Carotid atherosclerosis (n, %)	0.003	2.987 (1.466;6.086)	>0.001	3.51 (1.861;6.618)
Heart failure (n, %)	0.118	2.223 (0.817;6.045)	0.068	2.504 (0.934;6.717)
Coronary artery disease (n, %)	0.262	1.536 (0.726;3.248)		
Hypercholesterolemia (n, %)	0.216	1.579 (0.766;3.254)		
Diabetes (n, %)	0.209	1.515 (0.792;2.9)		
Past diagnosis of atherosclerosis (n, %)	0.751	0.889 (0.432;1.831)		

## Data Availability

Data available on request due to privacy restrictions.
